# Evaluating the clinical and cost effectiveness of a behaviour change intervention for lowering cardiovascular disease risk for people with severe mental illnesses in primary care (PRIMROSE study): study protocol for a cluster randomised controlled trial

**DOI:** 10.1186/s13063-016-1176-9

**Published:** 2016-02-12

**Authors:** David Osborn, Alexandra Burton, Kate Walters, Irwin Nazareth, Samira Heinkel, Lou Atkins, Ruth Blackburn, Richard Holt, Racheal Hunter, Michael King, Louise Marston, Susan Michie, Richard Morris, Steve Morris, Rumana Omar, Robert Peveler, Vanessa Pinfold, Ella Zomer, Thomas Barnes, Tom Craig, Hazel Gilbert, Ben Grey, Claire Johnston, Judy Leibowitz, Irene Petersen, Fiona Stevenson, Sheila Hardy, Vanessa Robinson

**Affiliations:** Division of Psychiatry, Epidemiology and Applied Clinical Research Department, Faculty of Brain Sciences, University College London, 6th Floor, Maple House, 149 Tottenham Court Road, London, W1T 7NF UK; Department of Primary Care and Population Health, Institute of Epidemiology and Health Care, University College London, Rowland Hill St, London, NW3 2PF UK; Division of Psychology and Language Sciences, Centre for Behaviour Change, Department of Clinical, Educational and Health Psychology, Faculty of Brain Sciences, University College London, 1-19 Torrington Place, London, WC1E 7HB UK; Human Development and Health Academic Unit, Faculty of Medicine, University of Southampton, University Rd, Southampton, SO17 1BJ UK; UCL Department of Applied Health Research, Institute of Epidemiology and Health Care, University College London, 1-19 Torrington Place, London, WC1E 7HB UK; School of Social and Community Medicine, University of Bristol, Canynge Hall, 39 Whatley Road, Bristol, BS8 2PS UK; The McPin Foundation, 32-36 Loman Street, London, SE1 0EH UK; Department of Medicine, Faculty of Medicine, Imperial College London, Commonwealth Building, Hammersmith Campus, Du Cane Road, London, W12 0NN UK; Institute of Psychiatry, Psychology & Neuroscience, Kings College London, 16 De Crespigny Park, London, SE5 8AF UK; Camden and Islington NHS Foundation Trust, 4th Floor, East Wing, St Pancras Hospital, 4 St Pancras Way, London, NW1 0PE UK; University of Northampton, Berrywood Hospital, Berrywood Drive, Upton, Northampton NN5 6UD UK; Rethink Mental Illness, 89 Albert Embankment, London, SE1 7TP UK

**Keywords:** Primary care, Severe mental illnesses, Cardiovascular risk

## Abstract

**Background:**

People with severe mental illnesses die up to 20 years earlier than the general population, with cardiovascular disease being the leading cause of death. National guidelines recommend that the physical care of people with severe mental illnesses should be the responsibility of primary care; however, little is known about effective interventions to lower cardiovascular disease risk in this population and setting. Following extensive peer review, funding was secured from the United Kingdom National Institute for Health Research (NIHR) to deliver the proposed study. The aim of the trial is to test the effectiveness of a behavioural intervention to lower cardiovascular disease risk in people with severe mental illnesses in United Kingdom General Practices.

**Methods/Design:**

The study is a cluster randomised controlled trial in 70 GP practices for people with severe mental illnesses, aged 30 to 75 years old, with elevated cardiovascular disease risk factors. The trial will compare the effectiveness of a behavioural intervention designed to lower cardiovascular disease risk and delivered by a practice nurse or healthcare assistant, with standard care offered in General Practice. A total of 350 people will be recruited and followed up at 6 and 12 months. The primary outcome is total cholesterol level at the 12-month follow-up and secondary outcomes include blood pressure, body mass index, waist circumference, smoking status, quality of life, adherence to treatments and services and behavioural measures for diet, physical activity and alcohol use. An economic evaluation will be carried out to determine the cost effectiveness of the intervention compared with standard care.

**Discussion:**

The results of this pragmatic trial will provide evidence on the clinical and cost effectiveness of the intervention on lowering total cholesterol and addressing multiple cardiovascular disease risk factors in people with severe mental illnesses in GP Practices.

**Trial registration:**

Current Controlled Trials ISRCTN13762819. Date of Registration: 25 February 2013.

Date and Version Number: 27 August 2014 Version 5.

## Background

People with severe mental illness (SMI), defined as schizophrenia, bipolar disorder and other nonorganic psychotic conditions, constitute 0.5 to 1 % of the United Kingdom population [1, 2]. The National Health Service (NHS) expenditure on mental health in 2012 to 2013 was £11.5 billion, higher than any other health category [3]. The predicted cost to the public health sector of schizophrenia is £7.6 billion, with the cost to society at £11.8 billion [4]. These disorders frequently present in the early to mid-twenties and have a major impact on health and social functioning. Whilst the employment rate for all adults aged 16 to 64 years in England is currently 71 %, recent estimates place the employment rate for people with schizophrenia between 5 and 15 % [5].

People with SMI are more likely to die prematurely from cardiovascular disease (CVD) than suicide [6–9]. CVD rates are falling in the general population but not for people with SMI [10, 11]. This widening health inequality is an NHS priority, emphasised in the National Institute for Health and Care Excellence (NICE) schizophrenia and bipolar guidelines [12, 13]. CVD is one of the most important physical health problems experienced by people with SMI. Those aged under 50 years are three times more likely to die from CVD, whereas those aged 50 to 75 years have a twofold increased risk [8]. These risks remain high after the effects of smoking and social deprivation are controlled for in the analysis.

There are likely to be multiple reasons for increased CVD in people with SMI. Research in general practice has demonstrated increased levels of cardiovascular risk factors including smoking, obesity, diabetes and dyslipidaemia in people with SMI compared to general practice controls [14]. This included raised total cholesterol and low density lipoprotein (LDL) cholesterol levels and lower levels of high density lipoprotein (HDL) cholesterol. Research has also found a higher proportion of people with SMI eat a diet higher in fat, lower in fibre and may be less likely to take even mild to moderate exercise [15]. A higher prevalence of increased CVD risk factors has been demonstrated at illness onset [16] and later in treatment [17].

The adverse effects of antipsychotic medication are a concern for both people with SMI and practitioners. The most effective and most commonly prescribed treatments are associated with weight gain and abnormalities of both lipid and glucose metabolism [18, 19]. The antipsychotics most strongly implicated are olanzapine and clozapine, but nearly all antipsychotics can have effects on appetite, weight gain and metabolism.

Most people with SMI are in contact with primary care and consult with General Practitioners (GPs) more frequently than people without SMI [9, 20], yet they are less likely to see a practice nurse [21]. NICE recommends routine annual screening for CVD risk factors for people with both schizophrenia and bipolar disorder in primary care [12, 13], and individuals prescribed antipsychotics should receive more frequent monitoring [22]. Clinics for CVD screening in other long-term conditions are routinely carried out by nurses in primary care. Training practice nurses to carry out physical health screening in people with SMI in England has the potential to increase the level of screening and lifestyle advice received by people with SMI [23, 24].

The UK primary care quality outcomes framework (QoF) remunerates GPs for providing an annual review to people with SMI. The required content of such reviews has varied over the last few years with glucose and lipid measurements being retired in 2014/15. Research has shown that if people with SMI are offered CVD screening, they are as likely to take up the offer as people without SMI [25, 26].

Although there is strong evidence for relatively high rates of CVD deaths and CVD risk factors in people with SMI, high quality evidence on CVD risk-reduction strategies is lacking. Strategies might include intervening earlier and more assertively than is currently recommended for the general population. Statins have been found to reduce CVD risk by up to 33 % in the general population [27]; however, we have limited evidence on the clinical effectiveness of existing treatments such as statins for people with SMI and whether they adhere to them. Statins have shown some promise in the short-term reduction of severe dyslipidaemia in people with SMI. An open, non-randomised study of statin therapy for 52 people with severe dyslipidaemia showed significant improvement in lipid levels at 12 weeks compared with 48 people not receiving statins [28]. Similarly, a pilot randomised controlled trial of Pravastatin identified a significant decrease in total cholesterol and LDL cholesterol in people with SMI; however, the effects were much smaller than in the non-randomised study described above, and this effect did not remain significant at 12 weeks [29].

Clinical trials of smoking cessation in people with SMI show small effects [30]. A Cochrane systematic review of reducing weight gain in people with SMI revealed little high quality evidence [31], with a handful of small studies showing modest effectiveness for pharmacological and behavioural interventions. A systematic review found that non-pharmacological interventions produced a 3.12 kg reduction in body weight [32]. Most studies, however, are short, lack statistical power, and many are not randomised. A longer, randomised effectiveness study is required to determine whether the impact of CVD risk reducing strategies can be maintained.

### Study objectives

The aim of the study is to test the clinical effectiveness and cost effectiveness of a behavioural intervention to lower CVD risk in primary care delivered to people with SMI.

The primary objective is to establish the effectiveness of the intervention in reducing total cholesterol over a 12-month period compared with treatment as usual (TAU).

The secondary objectives are to determine whether the intervention accomplishes the following:

1. reduces glycated haemoglobin (HbA_1c_), blood pressure, body mass index (BMI), waist circumference, total cholesterol/HDL cholesterol ratio, HDL cholesterol, and LDL cholesterol;
2. lowers cardiovascular risk scores;
3. increases physical activity, improves diet, reduces the number of cigarettes smoked and reduces alcohol intake;
4. increases the uptake of statin medications and adherence to statin regimens; and
5. increases satisfaction with services, wellbeing and quality of life.

We also aim to establish the cost-effectiveness of the intervention while considering both the costs of the intervention itself and other direct health care costs.

## Methods/Design

The study is a two arm cluster-randomised controlled trial in United Kingdom primary care services with people who have a diagnosis of SMI, are aged 30 to 75 years old and who have elevated CVD risk factors. GP practices will be randomised to provide either a 6-month CVD risk reduction intervention or usual care. The cluster design will decrease the likelihood of contamination by participants in the TAU arm receiving the benefits of the intervention through access to their practice nurse or healthcare assistant who has been trained in assertively reducing CVD risk for people with SMI.

### Recruitment

#### Recruitment of GP practices

Recruitment of 70 GP practices across both rural and urban areas of England will be coordinated in six waves. In the first wave, we will recruit eight GP practices in North London that will act as a pilot of the research procedures. Any problems will be considered and, if necessary, the trial will be modified for the remaining recruitment waves. Ten to 15 general practices will then be recruited per wave. GP practices will first be approached by local clinical research networks (CRNs) and asked to submit an expression of interest to the study team. Any interested practices will then be asked to complete a site feasibility questionnaire to ensure that they have a practice nurse or healthcare assistant who can assess patient eligibility and deliver the intervention if randomised, and a mental health register of 40 or more patients. A site initiation visit will then be arranged by the central study team. All staff involved in the study at the practice and the research nurse from the local clinical research network, who will be taking informed consent and collecting outcome data from participants, will be invited to attend this meeting.

#### Recruitment of participants

**Fig. 1:**
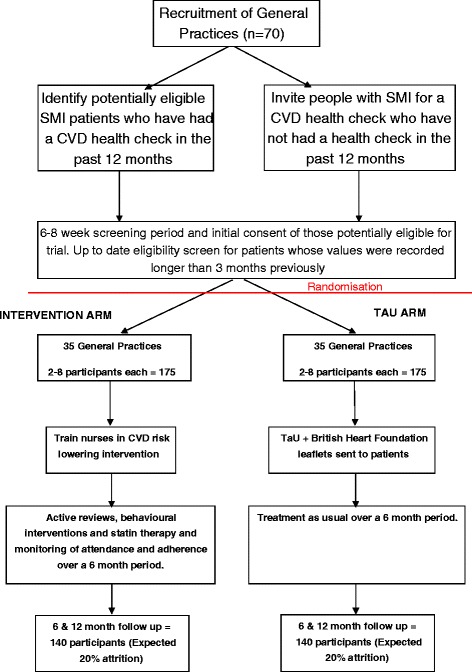
Flow chart of participant recruitment in the PRIMROSE trial

In each recruited GP practice, all people with a relevant diagnosis of SMI (see participant inclusion criteria) who have not had a CVD health check in the last 12 months will be invited to a screening appointment over an 8-week period with a practice nurse or healthcare assistant (HCA) who has been identified to work on the study. Individuals will be screened for CVD risk, to include current smoking status, lipid profile, blood pressure, HbA_1c_ and BMI. At the initial appointment, people with SMI will be asked by the practice nurse or HCA if they would like to receive further information about the study should they meet inclusion criteria. When screening results are received by the practice, if they are of immediate concern, the nurse/HCA will liaise with the GP. Otherwise, if an individual is eligible for the trial, the results will be handled by the nurse/HCA in a 6 to 8-week period, after the nurse/HCA has been informed of treatment allocation. The recruitment process is summarised in Fig. 1.

Practices will also search their electronic medical records to identify individuals who have had a health check in the last 12 months and who are potentially eligible for the study. These people will be sent a study invitation letter by the practice which includes a reply slip, study leaflet and freepost practice-addressed envelope. Practices will then follow-up non-responders by telephone to check if the information has been received and to determine whether or not the person is interested in participating. An up-to-date health check will be arranged either by the practice or the research nurse for all interested individuals for whom eligibility criteria were recorded more than 3 months previously to ensure that they are still currently eligible for the study.

#### Participant inclusion criteria

Inclusion criteria are adults on each participating GP Practice mental health register with a diagnosis of SMI (schizophrenia, persistent delusional disorder, schizoaffective disorder, bipolar affective disorder, psychosis, psychotic depression or other psychotic disorder), aged 30 to 75 years old with a total cholesterol level above and including 5.0 mmol/l OR raised total cholesterol/HDL cholesterol ratio above and including 4 AND one or more of the following risk factors:

1. BMI ≥ 30 kg/m^2^
2. Current smoker
3. Blood pressure ≥ 140 mm Hg systolic AND/OR ≥ 90 mm Hg diastolic
4. HbA_1c_ of 42 to 47 mmol/mol (6.0 to 6.4 %) and/or impaired fasting glucose (5.5 to 6.9 mmol/L)
5. Diagnosis of diabetes
6. Diagnosis of hypertension

Individuals must also be able to give written informed consent and give their initial consent to be contacted by a researcher.

#### Exclusion criteria

Exclusion criteria include the following:

1. Under acute psychiatric care (for example, currently a psychiatric hospital inpatient or under the care of a crisis team)
2. Primary diagnosis of an organic mental health problem and/or severe cognitive impairment
3. Life expectancy < 6 months (diagnosis of metastatic cancer or on the palliative care register)
4. Pre-existing CVD
5. Currently pregnant
6. Personality disorder or depression/anxiety without any psychotic features

We will not specifically exclude people who already receive statins if their lipids remain raised at the initial screening, since they still require monitoring and further risk reduction.

#### Consent and research assessments

Initial agreement to be contacted by a researcher from the local clinical research network will be obtained from individuals either at their screening appointment with the practice nurse/HCA or through the return of a reply slip to the GP practice sent to people who are already potentially eligible. If an individual does not respond to this letter, the GP practice will call them and ask whether they would be interested in receiving further information about the study from a research nurse. The researcher will then telephone each eligible and interested person to explain the study in detail and to determine whether they want to take part or not, answer any questions and arrange to obtain full written consent and a full baseline assessment. It will be explained that the individual is free to withdraw from the study at any time without having any effect on their care. If a participant is randomised to the intervention group and no longer wishes to attend the intervention appointments, he/she will still be invited to attend the follow-up assessments.

Written informed consent will be obtained from each participant before the baseline assessment begins. Baseline data will be collected by the researcher from the participant’s medical records and through interviews and participant self-complete questionnaires with all recruited participants at the start of the study. Outcome data will be collected at 6 and 12 months during the trial period. Participants will be allocated a unique study identification number, and data will be entered anonymously by researchers using a web-based system set up by Sealed Envelope [33]. It has range checks, consistency checks and for closed questions gives a number of options plus “other” where appropriate. Researchers who will be entering the data will have no access to the group allocation through this system. With these checks in place, there should not be any issues with illegal values or inconsistent data being entered, so necessary cleaning should be minimal. Data will be checked by the trial statistician before analysis and any problems reported to the trial manager, who will liaise with the researchers to rectify them as appropriate before data analysis.

Participants will be reimbursed £20 at baseline, £10 at the 6-month follow up and £20 at the 12-month follow-up to thank them for their time and to cover any transportation costs. Postcards will be sent after each data collection point to thank participants for their involvement in the study and remind them that follow-up will occur later in the year.

#### Blinding

The trial will not be blind because masking of the participants, practice nurses/HCAs and GPs to the treatment allocation is not possible. The primary outcome - total cholesterol - is objective, and researchers collecting outcome data will be blinded to participant allocation. The randomisation variable will be held separately to the main body of data and will be given to the statisticians and health economists without labels so they remain blind to allocation when they are ready to analyse the data.

#### Randomisation

GP practices will be randomised to intervention or to treatment as usual on a 1:1 basis by a statistician not otherwise involved in the programme based at the University College London PRIMENT Clinical Trials Unit. The trial manager will communicate the results of randomisation to the practices.

There should be the same number of GP practices recruited to the intervention and usual care. This will be achieved using block randomisation. The size of the blocks is at the discretion of the independent statistician, who will not divulge this to the trial management group, senior statistician, trial statistician or health economists until the allocation code is broken and the information is needed to write up the trial. Figure 1 demonstrates the recruitment pathway through the study.

#### Intervention arm

In the National Institute for Health Research (NIHR)-funded PRIMROSE programme we performed a range of work to inform the development of the PRIMROSE intervention. We consulted with experts in the fields of cardiovascular health, psychiatry and health psychology to ensure the intervention encompasses the most contemporary evidence, and that it was acceptable to people with SMI and health professionals. We have updated literature searches and run focus groups with stakeholders to ensure that the timing, content and delivery of the intervention are appropriate. We conducted focus groups with primary care nurses, people with SMI, carers, mental health experts and GPs [34]. We also benefited from a Lived Experience Advisory Panel (LEAP), which provided feedback on the intervention and associated materials [35].

Focus group interviews were underpinned by two complimentary theoretical models, the COM-B model [36] and the Theoretical Domains Framework (TDF) [37]; both have been used to identify influences on patient and health professional behaviour [38]. These findings were mapped to a theoretical framework of behaviour change, the Behaviour Change Wheel [36] to determine eight key behavioural strategies for practice nurses/HCAs to use when working with people with SMI to help reduce their CVD risk. These eight behavioural change strategies form the basis of the intervention manual and training programme and include goal-setting, making an action plan, recording progress, providing positive feedback, involving supportive others, reviewing progress, coping with setbacks and habit formation.

The training will take place over 2 days (with a 2-week gap in between each training session to enable rehearsal of skills) with opportunities for telephone supervision. Training day one includes the following sessions: i) the reasons for increased cardiovascular risk in people with severe mental illness; ii) severe mental illness symptoms, treatments and lived experience; iii) evidence on how the intervention manual and training programme were developed; iv) how to deliver the PRIMROSE intervention and use the PRIMROSE manual; and v) how to deliver the eight behavioural change strategies in consultations with recruited patients. Nurses and HCAs will then be asked to implement what they have learned with a recruited patient. Training day 2 is then an opportunity to feedback any problems, anything that went well and to practice and revisit the skills learned on training day 1.

The training will be delivered by members of the research team, a nurse with primary care and mental health expertise, and a lived experience trainer. The intervention has been documented in a written manual and will be distributed to practice nurses/HCAs. The nurse/HCA will be asked to record all of their work including appointment attendance, clinical measures and monitoring of health goals, and all consultations will be audio recorded. A sample of 20 % of the recordings will be transcribed and analysed to assess for fidelity to the manual using an adaptation of a reliable fidelity assessment method that has been developed for behavioural interventions including physical activity interventions [39].

Participants in the GP practices allocated to the intervention will be offered weekly to fortnightly appointments with the practice nurse/HCA over a period of 6 months. Progress with CVD risk reduction will be reviewed at each consultation. Appointments will decrease to monthly if satisfactory progress is made with reducing CVD risk. There will be flexibility over the delivery of the intervention depending on each individual’s preferences and needs; however, the intervention will include one or more of the following elements:

1. Setting and monitoring participant-led behavioural goals aimed at lowering CVD risk (for example, improving adherence to statins or other CVD risk lowering medications, improving diet, increasing physical activity, reducing smoking and/or alcohol consumption.
2. Use of a health plan to formulate a health goal and action plan and record progress with achieving the identified health goal.
3. Sign-posting to services such as smoking cessation or physical activity programmes.
4. Statin prescriptions to people whose CVD risk or lipids exceed recommended thresholds. The choice of statin will follow local and national treatment guidelines for statin prescribing. Current NICE guidance advises initial treatment with atorvastatin 20 mg unless there are drug interactions or contraindication [27]. Active monitoring of adherence to medication and response will be carried out.
5. Prescription of other medications such as nicotine replacement therapy for smoking cessation, anti-hypertensives for raised blood pressure or metformin for diabetes and weight loss. Again active monitoring of adherence, side effects and response will be carried out.
6. Involvement of carers and mental health key workers in monitoring adherence, supporting attendance at appointments and supporting a healthy lifestyle.

#### Treatment as usual arm

Practice nurses/HCAs in the standard care arm will not receive training. It will have been booked in their diaries, but they will be informed of allocation to the standard care arm, cancelling the training session. They will be sent British Heart Foundation leaflets [40] to distribute to participants. They will not be asked to review participants, to check adherence or arrange statin prescriptions. At best, we envisage that treatment as usual, will comprise of an invitation to attend an annual health check to screen for smoking, blood pressure, alcohol use and BMI; however, the intensity of usual care will vary depending on the patient’s health needs. Practice nurses/HCAs in the treatment as usual arm will be offered the opportunity to attend a training session once the intervention period is complete. Usual procedures of care for patients are permitted.

#### Measures

The primary outcome measure is total cholesterol at 12-month follow-up. A blood sample will be collected at baseline using usual GP practice equipment and procedures. Bloods are normally taken by the practice nurse or HCA and sent to the local NHS hospital Trust laboratory for analysis. Results are then received electronically through the participant’s medical record. The blood test will be repeated at 6 and 12-month follow up by a clinical research network nurse and processed using usual GP practice procedures described above.

The baseline data described below will be collected either directly from participant interviews/clinical measures or from the GP practice medical records.

#### Demographics

Demographic data including age, sex, marital status, employment status, ethnicity, Townsend deprivation score calculated using each participant’s postcode, mental healthcare worker details, whether or not the participant is on the Care Programme Approach (CPA), and whether they have a support worker or carer will be collected for each participant.

#### Clinical measures

Clinical measures including blood pressure, HbA_1c_ and lipids (HDL, Total/HDL ratio and LDL) will be measured using usual practice procedures. Other clinical measures include waist circumference, 10-year CVD risk scores (QRisk) [41], Framingham re-estimated on the UK population [42], and the PRIMROSE CVD risk prediction model [42], BMI, physical co-morbid conditions, and diagnosis of SMI confirmed by the GP practice medical record.

We will also ask participants at the baseline assessment if they would consent to providing a saliva sample for storage at UCL laboratories or collaborating centres in the UK and internationally for future genetic research. This will be used to determine whether there is any DNA variation relevant to mental health diagnosis and physical health outcomes. Participants will be given the opportunity to opt out of providing this saliva sample if they wish. It will not affect their entry into the trial.

#### Behavioural measures

CVD risk-lowering behaviours will be assessed through a validated self-report questionnaire on physical activity (IPAQ) [43] and validated researcher administered questionnaires on dietary intake (DINE) [44] and alcohol use (AUDIT-C) [45]. Each participant will also be asked their smoking status, and, if they are a current smoker; how many cigarettes they smoke a day.

The Morisky Scale of Adherence (MMS), a validated self-report questionnaire [46] will be completed twice at each time point by each participant. Participants will first be advised to complete the questionnaire while thinking about their psychiatric medications (for example, antipsychotics and mood stabilisers) and secondly in relation to any CVD risk-lowering medications they are currently prescribed (for example, statins, anti-hypertensives and/or stop smoking aids).

#### Quality of life and wellbeing

Quality of life will be measured through the EQ-5D-5 L, a validated self-report questionnaire [47], Well-being will be assessed using the self-report Warwick Edinburgh Mental Wellbeing Scale (WEMWEBS) [48].

#### Economic measures

Economic measures will be collected from the medical records at baseline and 12-month follow-up, including medications used in the 12-months prior to baseline and any new prescriptions during the study follow-up period. General and psychiatric hospital admissions, Accident and Emergency (A&E) contacts, outpatient visits, primary care contacts and community service use in the previous 12 months will all be collected from the medical records. In addition, participants will self-complete an adapted Client Service Receipt Inventory (CSRI) to collate additional information on service use, which is unlikely to be recorded in the medical records (for example, informal care arrangements, living situation, CVD risk-lowering service use external to the NHS, such as weight-management programmes, gym and leisure activities, and community service use [49].

#### Follow-up assessments

Follow-up assessments will be conducted at 6 and 12 months and will include all clinical measures, behavioural measures, quality of life and economic measures. At 12 months, we will additionally collect information on adherence to the PRIMROSE intervention appointments and client satisfaction using the Client Satisfaction Questionnaire - (CSQ) [50].

A table summarising all outcome measures and data collection time points can be found in Table 1.

**Table 1:** Summary of baseline and follow-up measures

Measure	Source data	0 mth	6 mth	12 mth
1. Clinical measures				
Total cholesterol (mmol/l)	Blood test	✓	✓	✓
HDL, LDL, Total cholesterol/HDL ratio (mmol/l)	Blood test	✓	✓	✓
HBA1c (mmol/mol, %)	Blood test	✓	✓	✓
Systolic and diastolic blood pressure (mm/hg)	Clinical assessment	✓	✓	✓
2. Anthropometric measures				
BMI (kg/m^2^)	Clinical assessment	✓	✓	✓
Waist circumference (cm)	Clinical assessment	✓	✓	✓
3. Behavioural measures				
Alcohol intake (AUDIT-C)	Researcher administered questionnaire	✓	✓	✓
Smoking status	Researcher administered questionnaire	✓	✓	✓
Diet (DINE)	Researcher administered questionnaire	✓	✓	✓
4. Medical records.				
Active physical health conditions	Medical records	✓		✓
Service use (inpatient hospital stays, outpatient appointments, accident and emergency attendances, community service use, health checks and action plans)	Medical records	✓		✓
Prescribed medications	Medical records	✓		✓
CVD risk scores	Medical records	✓		✓
Scheduled appointments not attended	Medical records	✓		✓
5. Patient questionnaires				
Service use: Employment, Housing, Health and Leisure (EHHaL)	Patient self-report questionnaire	✓	✓	✓
International Physical Activity Questionnaire (IPAQ)	Patient self-report questionnaire	✓	✓	✓
Quality of life: EQ-5D-5 L	Patient self-report questionnaire	✓	✓	✓
Wellbeing: The Warwick-Edinburgh Mental Well-Being Scale (WEMWBS)	Patient self-report questionnaire	✓		✓
Morisky Scale of Adherence (MMS): Psychiatric medications	Patient self-report questionnaire	✓	✓	✓
Morisky Scale of Adherence (MMS): CVD preventative medications	Patient self-report questionnaire	✓	✓	✓
Client Satisfaction Questionnaire - CSQ	Patient self-report questionnaire	✓	✓	✓

### Statistics and data analysis

#### Sample size

A total of 350 people from 70 GP practices will be recruited in to the study. In determining the size of the trial, we considered 1) important effect sizes, 2) size of clusters, and 3) attrition.

#### Effect size

Our primary outcome is total cholesterol. Two community studies of UK adults with SMI reveal mean total cholesterol levels of 5.4 mmol/l (standard deviation (SD) 1.3) [14] and 5.7 mmol/l (SD 1.4) [51].

We consider an effect size of 0.4 SDs difference in cholesterol, between the two trial arms, to be the minimum clinically important difference. Statins usually have far greater effect sizes, approaching two SDs [52], however in this effectiveness trial, we are comparing a behavioural intervention with treatment as usual, so cannot expect effects as large as this. Based on a two-sample t-test, to detect a difference of 0.4 x (1.3 mmol/l) requires 132 participants per arm, with 90 % power and 5 % significance level.

#### Sample size inflation for clustering and attrition

To account for the cluster effect, we have assumed that five participants on average will be recruited per practice. Dropout rates in the SMI trials are <20 % at 12 months [30, 53]. Using an intra-class correlation coefficient of 0.02 for trials in primary care [54] and an average cluster size of four participants per practice (after allowing for 20 % attrition) yields 140 participants per arm. Inflating this figure for 20 % attrition and rounding up results equates to a total of 350 participants and 70 GP practices for the trial.

#### Statistical analysis

All analyses will be on an intention-to-treat basis. Statistical analyses will be carried out using Stata [55]. We will produce summary statistics for all variables, both overall and by randomised group. Summary statistics for continuous variables will be mean or median, (with SD, or interquartile range) as appropriate, and for categorical variables, frequency and percentage within each category.

The primary outcome will be analysed using a linear random effects regression model adjusting for the baseline cholesterol, whilst accounting for clustering as a random effect (GP practice). Results will be presented as mean difference between randomised arms with 95 % confidence interval and *P* value.

Appropriate analogous random effects regression models will be used to analyse the secondary outcomes.

For both the primary and continuous secondary outcomes, the residuals will be checked for normality. If they are not normally distributed, outcomes may be transformed. If it is necessary to categorise outcomes, it will be done with clinical input.

Complete case analyses will be the main analyses from this study. Bias due to missing data will be investigated. A supportive analysis will include adjusting for predictors of missingness that are related to outcome, if necessary, and adjusting for factors that have baseline imbalances that are related to the outcome. If the proportion of non-adherence is greater than 5 %, an appropriate analysis accounting for non-adherence will be considered. Those in the treatment-as-usual practices will be assigned an adherence score equivalent to no adherence to the intervention. If it is appropriate to carry out this analysis, it will be done once the statistician has been unblinded to randomised group. Results from the analysis of secondary outcomes and supportive analyses will be presented as estimates with 95 % confidence intervals.

#### Economic evaluation

The aim of the economic evaluation is to report the mean incremental cost of the PRIMROSE intervention compared to treatment as usual in the reduction of CVD risk in people with SMI, from the NHS perspective in the primary analysis and from a societal perspective in a secondary analysis. The primary effectiveness outcome, as recommended by NICE, will be quality-adjusted life years (QALYs) [56]. Responses to the EQ-5D-5 L questionnaire collected during the trial will be used in conjunction with EQ-5D-5 L specific valuation sets [47] to calculate utility scores over a time horizon of 1 year. Baseline differences in utility scores will be adjusted for using regression analysis. The cost of the intervention will include the cost of the printed materials, training, clinical tests and other consumables used during the intervention and nurse/HCA time spent delivering the intervention. The cost-components that will be included in the health care perspective analysis for arms of the trial include new prescriptions during the study follow-up period, general and psychiatric hospital admissions, A&E contacts; outpatient visits, primary care contacts and community service use. Informal care arrangements, changes in living situation, carer costs, and out-of-pocket costs for services such as weight-management programmes, gym and leisure activities, smoking cessation and community service use [48] will be included in the societal perspective analysis. Unit costs will be obtained from the Personal Social Services Research Unit (PSSRU) [57], reference costs [58], British National Formulary (BNF) [59] and other national sources of costing information. Cost-effectiveness will be reported as the mean incremental cost per QALY gained of the PRIMROSE intervention versus treatment as usual. All cost-effectiveness analyses will conform to the recommended methodology [56]. Bootstrapping will be used to derive 95 % confidence intervals for mean cost and QALYs for the two trial groups, 95 % confidence intervals around the incremental cost-effectiveness ratios and cost-effectiveness acceptability curves and cost-effectiveness planes. The analyses will be subjected to one-way and two-way sensitivity analyses to test any assumptions made in the analysis. Trial results will also be used to update a long-run CVD cost-effectiveness model, extrapolating costs and consequences to a 10-year time horizon using discrete event-simulation.

### Research governance

We will work with the Clinical Research Networks (CRNs) to identify expressions of interest from GP practices to take part in the study.

A trial management subgroup will meet monthly at the start of the trial to monitor progress. At trial month 3, the group will review the effectiveness of research procedures in the first wave of practices and amend the protocol if necessary. Meetings will decrease to 3-monthly from month 18 of the trial onwards. There will also be an externally chaired trial steering committee meeting twice per year.

The trial will be fully registered and managed through the PRIMENT clinical trials unit (http://www.ucl.ac.uk/pcph/priment), which specialises in trials in mental health and primary care. PRIMENT will oversee the design, randomisation of practices, web-based data entry, all trial monitoring and the analysis plan. Data management will also be overseen by the PRIMENT clinical trials unit, with data being held for a minimum of 20 years from completion of the study according to University College London requirements.

### Adverse events

All GP practices and research nurses will be trained in reporting adverse events procedure. If an event occurs, they will be instructed to telephone the trial manager within 24 hours of becoming aware of the event. They will then be asked to complete an adverse event form and submit this to the trial manager. Adverse events will be assessed by the chief investigator, considered at trial management team meetings and reported to the study sponsor.

### Ethics approval

Ethics approval was obtained from the City Road and Hampstead Research Ethics Committee (Reference No: 12/LO/1934, approval granted 10 January 2013). Local NHS approvals are being obtained before the start of each recruitment wave from the following research and development (R&D) departments: North and Central London research consortium (Noclor), Wessex primary care research support service, Northamptonshire research and development service, North of England commissioning support unit, Avon primary care collaborative, Lincolnshire community health services NHS trust, clinical research network: North West Coast clinical commissioning group, Norfolk & Suffolk primary & community care research office, Leicester City clinical commissioning group, South London clinical research network, West Midlands Primary Care RM&G business support service and clinical research network: West Midlands, research support team. The trial has been allocated an International Standard Randomised Controlled Trials Number (ISRCTN13762819).

### Study sponsorship

Camden and Islington NHS Foundation Trust is the trial sponsor.

## Discussion

The PRIMROSE intervention has been developed using evidence from the literature, focus groups and workshops with key stakeholders. The intervention is underpinned by a theoretical framework [36], which was applied to the development work findings in order to select simple behaviour change strategies that can be used by practice nurses or HCAs, with minimal training, and incorporated into time-limited primary care consultations.

Existing reviews identify a need for interventions that tackle multiple cardiovascular risk factors in people with SMI [60]. Many of the interventions that have been tested in this group have been intensive behavioural interventions for weight loss or smoking cessation, delivered in secondary care settings by research therapists [61, 62]. The PRIMROSE study is a pragmatic trial with the intervention being delivered by a practice nurse/HCA at each participant’s GP practice. It may be possible to implement this model as part of routine clinical primary care, for example, in a UK setting as part of an Enhanced Service Model [63].

Offering cardiovascular risk lowering interventions to people with severe mental illnesses may provide an opportunity for early intervention and prevention before cardiovascular events occur. Physical health checks for people with SMI can be limited and are often opportunistic [33]. The PRIMROSE intervention seeks to address this issue, offering intensive consultations over a 6-month period that complement the clinical skills of practice nurses or HCAs to manage multiple CVD risk factors.

The results will assess the impact of the intervention on total cholesterol at 12-month follow-up. The impact of the intervention on secondary outcomes of health behaviours, quality of life, well-being, medication adherence, service use and medication prescriptions will be measured. The cost effectiveness of the intervention will also be determined.

### Dissemination

We will disseminate the results of the study through social media, the Lived Experience Advisory Group and through summary reports of findings to all key stakeholders involved in the study.

### Trial status

The trial is currently ongoing.

## Abbreviations

A&E: Accident and Emergency
AUDIT-C: Alcohol Use Disorders Identification Test Consumption
BMI: Body Mass Index
BNF: British National Formulary
COM-B model: Capability Opportunity Motivation – Behaviour model
CRN: Clinical Research Network
CSQ: Client Satisfaction Questionnaire
CSRI: Client Service Receipt Inventory
CVD: Cardiovascular Disease
DINE: Dietary Instrument for Nutrition Education
DNA: Deoxyribonucleic Acid
EHHaL: Employment Housing, Health and Leisure Questionnaire
GP: General Practitioner
HbA_1c_: Glycated Haemoglobin
HCA: Healthcare Assistant
HDL: High Density Lipoporotein cholesterol
IPAQ: International Physical Activity Questionnaire
LDL cholesterol: Low Density Lipoprotein cholesterol
LEAP: Lived Experience Advisory Panel
MMS: Morisky Scale of Adherence Questionnaire
NHS: National Health Service
NICE: The National Institute for Health and Care Excellence
NIHR: National Institute for Health Research
PGfAR: Programme Grants for Applied Research
PSSRU: Personal Social Services Research Unit
QALY: Quality-Adjusted Life Year
QoF: Quality Outcomes Framework
SD: Standard Deviation
SMI: Severe Mental Illness
TAU: Treatment as Usual
TDF: Theoretical Domains Framework
WEMWEBS: Warwick Edinburgh Mental Wellbeing Scale
